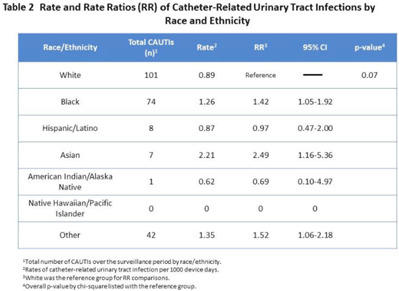# Racial disparities in rate of central-line–associated bloodstream infections and catheter-associated urinary tract infections

**DOI:** 10.1017/ash.2022.216

**Published:** 2022-05-16

**Authors:** Erin Gettler, Jessica Seidelman, Jay Krishnan, Naseem Alavian, Ibukun Kalu, Melissa Campbell, Sarah Lewis, Deverick Anderson, Becky Smith

## Abstract

**Background:** Racial and ethnic disparities in healthcare access, medical treatment, and outcomes have been extensively reported. However, the impact of racial and ethnic differences in patient safety, including healthcare-associated infections, has not been well described. **Methods:** We performed a retrospective review analyzing prospectively collected data on central-line–associated bloodstream infection (CLABSI) and catheter-associated urinary tract infection (CAUTI) rates per 1,000 device days. Data for adult patients admitted to an academic medical center between 2018 and 2021 were stratified by 7 racial and ethnic groups: non-Hispanic White, non-Hispanic Black, Hispanic/Latino, Asian, American Indian/Alaska Native, Native Hawaiian/Pacific Islander, and othe. The “other” group was composed of bi- or multiracial patients, or those for whom no data were reported. We compared the CLABSI and CAUTI rates between the different racial and ethnic groups using Poisson regression. **Results:** Compared to non-Hispanic White patients, the rate of CLABSI was significantly higher in non-Hispanic Black patients (1.27; 95% CI, 1.02–1.58; *P* < .03) and those in the “other” race category (1.79; 95% CI, 1.39–2.30; *P* < .001, respectively), and these trends increased in Hispanic/Latino patients (Table 1). Similarly, Black patients had higher rates of CAUTI (1.42; 95% CI, 1.05–1.92; *P* < .02), as did Asian patients (2.49; 95% CI, 1.16–5.36; *P <* .02), and patients in the “other” category (1.52; 95% CI, 1.06–2.18; *P* < .02) (Table 2). **Conclusions:** Racial and ethnic minorities may be vulnerable to a higher rate of patient safety events, including CLABSIs and CAUTIs. Additional analyses controlling for potential confounding factors are needed to better understand the relationship between race or ethnicity, clinical management, and healthcare-associated infections. This evaluation is essential to inform mitigation strategies and to provide optimum, equitable care for all.

**Funding:** None

**Disclosures:** None

**Table:**
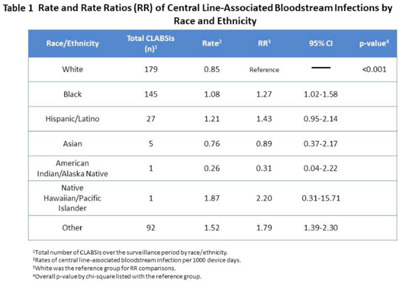

**Table:**